# Towards New Strategies: Case Report and Review of the Literature—Effective Use of JAK Inhibitor Baricitinib in a 4-Year-Old Boy with Anti-MDA5 Antibody-Positive Juvenile Dermatomyositis

**DOI:** 10.3390/jcm15020709

**Published:** 2026-01-15

**Authors:** Oana Buzoianu, Özlem Satirer, Jasmin B. Kuemmerle-Deschner, Christiane Reiser

**Affiliations:** 1 Division of Pediatric Rheumatology, Department of Pediatrics and Autoinflammation Reference Center Tuebingen, University Hospital Tuebingen, 72076 Tuebingen, Germany; oana.buzoianu@med.uni-tuebingen.de (O.B.); kuemmerle.deschner@uni-tuebingen.de (J.B.K.-D.); christiane.reiser@med.uni-tuebingen.de (C.R.); 2 Department of Pediatrics, Landeskrankenhaus Bregenz, Carl-Pedenz-Str. 2, 6900 Bregenz, Austria

**Keywords:** juvenile dermatomyositis, MDA-5 antibodies, interferon signature, JAK inhibitors

## Abstract

Juvenile dermatomyositis (JDM) is a rare, idiopathic autoimmune disorder characterized by inflammation of both muscle and skin, with a significant contribution from the interferon (IFN) pathway in its pathogenesis. Here, we present the case of a 4-year-old boy with JDM who tested positive for Mi2-α and MDA5 antibodies and showed combined muscle and skin involvement. In view of his markedly elevated IFN signature, the Janus kinase (JAK) inhibitor baricitinib was introduced very early as a targeted steroid-sparing agent in addition to standard immunosuppressive therapy. The patient experienced marked clinical improvement, with resolution of skin lesions, normalization of MDA5 antibodies, and a pronounced reduction in the IFN signature. This case highlights the potential efficacy of JAK inhibition in managing JDM with a high IFN signature and supports a mechanism-based, interferon-targeted treatment approach, in line with emerging evidence in refractory JDM. Further studies are warranted to define the role of JAK inhibitors in the treatment of JDM.

## 1. Introduction

Juvenile dermatomyositis (JDM) is a rare, idiopathic autoimmune condition characterized by inflammation of muscles and skin, which can lead to significant morbidity and, in severe instances, mortality [1]. Although the exact cause of JDM remains unknown, it is generally assumed that genetic predisposition, in combination with environmental triggers, initiates the autoimmune response [2]. Gene expression studies in both adult and juvenile dermatomyositis (DM and JDM) have consistently shown upregulation of interferon (IFN)-regulated genes, indicating that the IFN pathway plays a central role in the disease development [3,4,5]. In particular, high expression of type I and type II IFN-inducible genes in the myocytes of DM patients has been linked to increased inflammation and tissue repair mechanisms [4]. The strong association between IFN signaling and disease activity in DM, along with the crucial involvement of Janus kinases (JAKs) in IFN signal transduction, suggests that targeting the JAK-STAT pathway may represent a promising therapeutic strategy [6].

Janus kinase inhibitors exert their immunomodulatory effects by blocking intracellular signaling downstream of multiple cytokine receptors, particularly those involved in type I interferon signaling. Binding of type I interferons to their receptors activates JAK1 and TYK2, leading to phosphorylation of STAT proteins and induction of interferon-stimulated genes [7]. In dermatomyositis, persistent activation of this pathway results in a prominent type I interferon gene signature and contributes to chronic inflammation and tissue damage. By inhibiting JAK activity, JAK inhibitors suppress interferon-driven inflammatory signaling. This mechanism is especially relevant in anti-MDA5–associated disease, which is characterized by a strong type I interferon signature and severe systemic manifestations, including interstitial lung disease [8].

In JDM, the presence of specific autoantibodies is associated with distinct clinical phenotypes. Notably, MDA5 antibodies are correlated with a higher risk of skin ulcers, arthritis, and interstitial lung disease (ILD), often in the context of a predominantly amyopathic or hypomyopathic disease course [8]. ILD related to MDA5 antibodies is particularly concerning due to its treatment resistance and high mortality [9]. In such cases, conventional treatments may be ineffective, requiring additional therapies such as rituximab, TNF-alpha inhibitors, or cyclophosphamide [10,11,12]. Given the crucial role of the JAK-STAT pathway in JDM pathophysiology, JAK inhibitors have emerged as a promising therapeutic option [13,14]. Although several case reports have described the effectiveness of JAK inhibitors in refractory JDM, the current evidence remains limited, and further studies are needed to confirm these observations.

This case report describes the successful use of JAK inhibition in a 4-year-old boy with positive Mi2-alpha and MDA5 antibodies, presenting with both muscle and skin involvement.

## 2. Case Report

**Figure 1:**
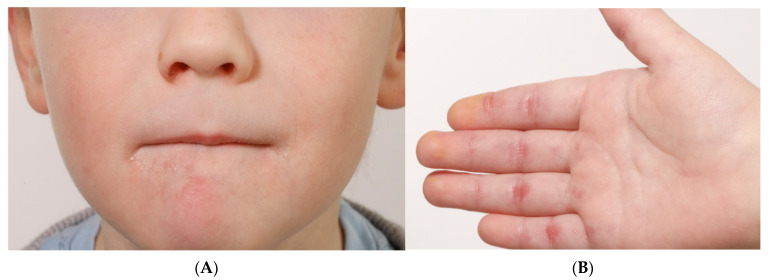

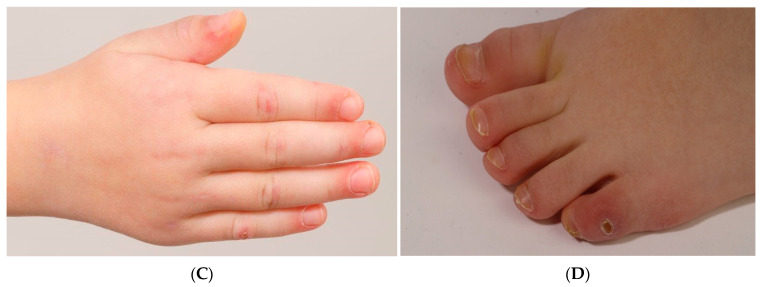
(**A**): Patchy, pale erythema on both cheeks and chin. (**B**): Peeling and erythema on the palmar sides and flexor surfaces of the hands. (**C**): Periungual erythema, thickening of the nail fold, and early-stage Gottron papules on the extensor surfaces of the fingers. (**D**): Livid macula with superimposed scaly plaque over ulceration on the right foot.

A previously healthy 4-year-old boy was admitted to the hospital, presenting with a history of heliotrope rash, bilateral eyelid edema, and vesicular lesions primarily localized to the oral cavity. Over time, the symptoms progressed, leading to the development of crusted lesions on the hands, feet, nose, and ears (Figure 1). Over the subsequent weeks his condition deteriorated.

His mother reported progressive fatigue and lethargy, accompanied by knee and ankle pain that led to refusal to walk. Additionally, he had complained of abdominal pain during the summer months, which prompted a trial of a lactose-free diet, which did not alleviate his symptoms.

On examination, there was marked muscle pain and weakness, symmetrically affecting both the upper and lower limbs, including the wrist extensors, neck flexors, gluteal muscles, and hip flexors. Skin findings included Gottron’s papules on the extensor surfaces of the fingers, knees, and elbows, as well as nodules suggestive of vasculitis in acral areas. Inflammatory changes were also observed in the skin and subcutaneous tissue, together with hepatosplenomegaly and lymphadenopathy, but there was no pulmonary involvement. Baseline MMT was 69/80, CMAS 33/52. Nailfold capillaroscopy was performed but inconclusive due to shallow nail fold.

Laboratory investigations detected Mi2-α and MDA-5 antibodies. semi-quantitative measurements of autoantybodies were performed with detection of Mi2 (Elisa), Mi2-α (immunoblot), MDA-5 (immunoblot). Muscle enzymes, including creatine kinase or aldolase were within normal range, with no leucocytosis and no elevated CRP or ESR.

The skin biopsy showed a discrete interface reaction with increased plasmacytoid dendritic cells and discrete dermal toluidine deposits.

Although the patient had anti-MDA5 antibodies, which raised concerns for potential pulmonary complications, pulmonary involvement was carefully excluded. Imaging studies included chest X-ray and whole-body magnetic resonance imaging with pulmonary sequences; no pathological findings were detected. In addition, pulmonary function tests, including diffusion capacity for carbon monoxide (DLCO), were within normal limits at diagnosis and remained normal during follow-up. No clinical or radiological signs of interstitial lung disease were observed.

**Figure 2:**
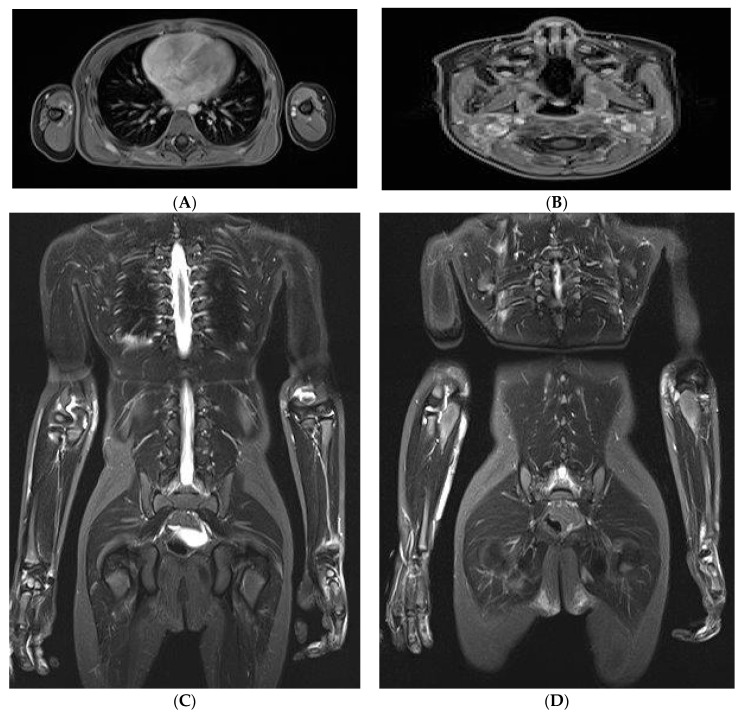
Whole-body MRI imaging: multiple muscular edemas and contrast enhancement consistent with myositis, adjacent to the scapula (**A**), cervical region (**B**), arthritis of elbow, hand (**C**), and multiple cutaneous and subcutaneous edematous and contrast-enhancing inflammatory changes with maximum intensity in the forearms (**D**). Images courtesy of the Department of Pediatric Radiology, University hospital Tuebingen.

MRI imaging revealed multiple muscular edemas and contrast enhancement consistent with myositis, with a maximum intensity bilaterally in the gluteus maximus muscles, to a lesser extent also in the adductor muscles, adjacent to the scapula (Figure 2A), and on the right side of the cervical region (Figure 2B). Synovitis of the elbow joints (Figure 2C), hands, knees, and ankles, the latter also showing tenosynovitis. Multiple cutaneous and subcutaneous edematous and contrast-enhancing inflammatory changes with maximum intensity in the forearms (Figure 2D) and gluteal regions. Additional smaller foci are noted, for example, along the left proximal anterior tibial margin and in the cervical region.

Clinical and radiological findings, along with auto-antibodies and conclusive histopathological findings confirmed the diagnosis of JDM.

Standard therapies with IVIG (2 g/kg), one cycle of methylprednisolone pulse steroids (30 mg/kg), and hydroxychloroquine 200 mg every other day were initiated while awaiting the interferon-signaling results. The interferon signature was measured by assessing the expression of interferon-stimulated genes (ISGs) in peripheral blood mononuclear cells (PBMCs). The following six ISGs were analyzed: IFI27, IFI44, IFI44L, IFIT1, ISG15, RSAD2, and SIGLEC1.

**Figure 3:**
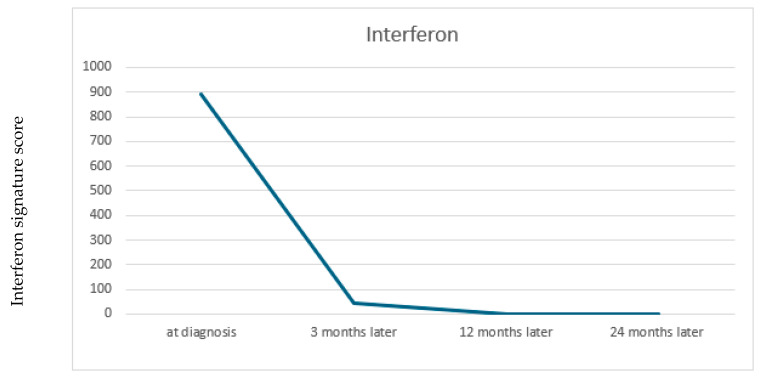
IFN signature score elevation and improvement. The patient had a highly elevated interferon (IFN) signature (score: 892.89; reference range: <12.49). Expression of six interferon-stimulated genes (ISGs) was analyzed: *IFI27*, *IFI44*, *IFI44L*, *IFIT1*, *ISG15*, *RSAD2*, and *SIGLEC1*. The IFN signature markedly improved and became negative one year after diagnosis.

Gene expression was normalized to GAPDH and compared to the mean expression of 10 healthy controls. Quantitative RT-PCR (TaqMan) was used for analysis, and results were expressed as mean fold change (relative mRNA expression) ± SEM from at least three measurements. Interferon signature was highly elevated (892.89, Ref. range: <12.49), (Figure 3). which led us to explore treatment options targeting the interferon pathway. Methotrexate was not introduced, as baricitinib was selected early on as the primary steroid-sparing agent in addition to corticosteroids, IVIG, and hydroxychloroquine. Off-label JAK inhibitor treatment with Baricitinib (2 mg/day, adapted to body weight), was introduced after 6 weeks after initial admission to target interferon signaling, particularly in the context of MDA5 antibodies [13]. No further steroids were administered from then on.

Baricitinib was selected over other JAK inhibitors based on its JAK1/JAK2 inhibition profile, its relevance in interferon-mediated diseases, and our center’s prior clinical experience with baricitinib in other pediatric rheumatologic conditions, particularly polyarticular juvenile idiopathic arthritis, where it has demonstrated good tolerability and manageable safety in routine clinical practice.

Treatment duration was guided by sustained clinical response and laboratory improvement, with therapy continued under close clinical and laboratory monitoring in the absence of established discontinuation algorithms.

**Figure 4:**
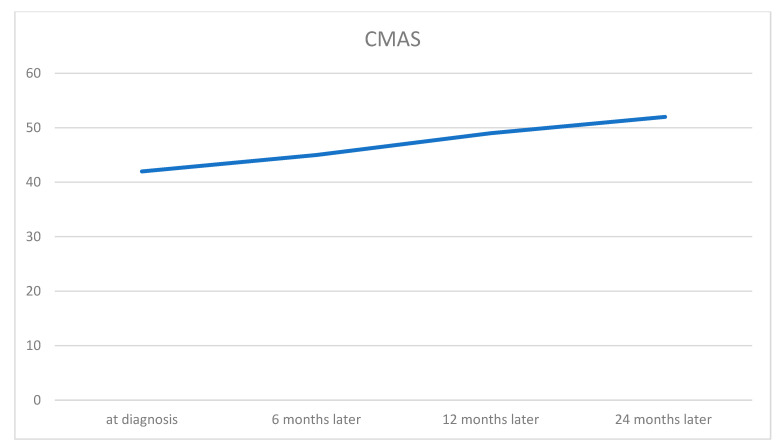
Improvement in CMAS over time. The patient’s CMAS score increased progressively from diagnosis to 24 months, indicating continuous improvement in muscle strength and function. CMAS was assessed at diagnosis, and at 6, 12, and 24 months of follow-up. CMAS: Childhood Myositis Assessment Scale.

The patient experienced substantial clinical improvement on Baricitinib treatment, remaining on a stable dose of 2 mg. His CMAS score increased significantly from 42/52 upon admission to 52/52 after 24 months (Figure 4), while MDA5 antibodies and IFN signature were no longer detectable. Over time, the skin lesions healed, with complete resolution of ulcerations and minimal scars remaining. There were no recurrences of skin lesion recurrence or clinical manifestations of calcinosis.

The patient remains in sustained stable remission without any signs of disease activity on continuous Baricitinib therapy.

## 3. Discussion

This case demonstrates that Janus kinase (JAK) inhibition can be an effective therapy for patients with JDM, particularly those with a high interferon signature and MDA5 antibodies. The addition of the JAK inhibitor to the therapeutic regimen led to a rapid resolution of symptoms and disease control. This decision was guided by the markedly elevated interferon signature, the presence of MDA5 antibodies, and accumulating evidence supporting JAK inhibition as a targeted approach in JDM.

The primary goals in JDM therapy are to control inflammation, restore muscle strength, and prevent long-term sequelae [12,15]. Depending on disease severity, individualized therapeutic strategies should be employed. While corticosteroids and methotrexate remain first-line treatments, complications such as calcinosis, lipodystrophy, interstitial lung disease (ILD), and joint contractures pose significant management challenges. In refractory cases, disease-modifying anti-rheumatic drugs (DMARDs) beyond methotrexate, biologic agents, and small molecules are utilized [12,13,14].

Our patient demonstrated an elevated type I interferon (IFN) signature characterized by increased expression of *IFI27*, *IFI44L*, *IFIT1*, and *RSAD2* (*viperin*), a validated whole-blood marker set reflecting systemic IFN-I pathway activation. This transcriptional signature has been shown to correlate with both muscle and skin disease activity in dermatomyositis [16,17]. In particular, the IFN signature decreases with effective therapy, including Janus kinase (JAK) inhibition, underscoring its value as a pharmacodynamic biomarker. In parallel, recent studies have identified circulating IFN-stimulated chemokines—*CXCL10* (IP-10), *CXCL11* (I-TAC), and *galectin-9* (Gal-9)—as biomarkers that correlate with disease activity and longitudinal improvement in JDM. Monocyte expression of SIGLEC1 (CD169) also mirrors IFN-pathway activation and has emerged as a sensitive biomarker for both disease activity and therapeutic monitoring. Notably, Veldkamp et al. (2025) demonstrated that SIGLEC1 expression can serve as an in vitro biomarker of JAK-inhibitor efficacy in JDM, directly linking IFN-pathway activity with target engagement [18]. Collectively, these findings highlight how IFN-pathway biomarkers—including gene-expression signatures, inducible proteins, and SIGLEC1—can inform and rationalize targeted therapy in JDM. The favorable clinical response observed in our patient following JAK-inhibitor therapy supports this biologically guided approach and underscores the potential value of implementing IFN biomarker testing more broadly in clinical practice.

A comprehensive review of the literature was conducted using PubMed to identify studies reporting the use of JAK inhibitors in juvenile dermatomyositis (JDM). The search was performed using the keywords “JDM” and “JAK inhibitors,” covering publications from 2018 to 2025. A total of 27 reports and studies were identified, including cases treated with various JAK inhibitors, such as ruxolitinib, baricitinib, and tofacitinib. Overall, approximately 270 patients were reported to have received JAK inhibitor therapy, highlighting the emerging role of these agents in the management of refractory or severe JDM (Table 1). In addition, baricitinib has an established regulatory approval in pediatric populations, being indicated for atopic dermatitis and juvenile idiopathic arthritis from 2 years of age [19]. This existing pediatric safety and efficacy profile further supports the feasibility of baricitinib use in children and reinforces its potential as a therapeutic option in refractory juvenile dermatomyositis.

This growing body of evidence highlights the therapeutic potential of JAK inhibitors in JDM. Subsequent studies have specifically examined their efficacy in refractory cases, focusing on agents such as ruxolitinib, baricitinib, and tofacitinib. Aeschlimann et al. (2018) reported significant clinical improvement in a 13-year-old girl treated with ruxolitinib, with disease inactivity achieved at the 12-month follow-up [11]. Similarly, Heinen et al. (2021) observed marked recovery of muscle strength and reduction in inflammatory markers in a 14-year-old patient with anti-NXP2-positive JDM [20]. Larger studies, such as that by Huang et al. (2023), confirmed significant improvement of rash, muscle strength, and laboratory markers in 128 JDM patients receiving JAK inhibitors [21]. Notably, Ding et al. (2021) found that 96% of JDM patients exhibited rash improvement, with 66.7% achieving complete resolution of the rash, while muscle strength recovery was observed in 40% [22].

JAK inhibitors have also demonstrated efficacy in severe cases complicated by interstitial lung disease (ILD). Chan Ng et al. (2022) reported successful remission induction and maintenance with tofacitinib in pediatric JDM with rapidly progressive ILD [23]. Kaplan et al. (2023) highlighted clinical improvement and reduced interferon-alpha levels in anti-MDA5-positive JDM patients with ILD and cardiac involvement [24].

Calcinosis-associated JDM also appears responsive to JAK inhibition. Agud-Dios et al. (2022) described significant softening of calcium deposits and regained mobility in a 5-year-old patient treated with baricitinib [25]. Mastrolia et al. (2023) further supported the efficacy of baricitinib in refractory JDM complicated by calcinosis [26] (Table 1).

The safety profile of JAK inhibitors in JDM remains favorable, with most studies reporting good tolerability and minimal adverse effects. Aeschlimann et al. (2018) and Papadopoulou et al. (2019) observed no major adverse events in their respective patients treated with ruxolitinib and baricitinib, while corticosteroid tapering was achieved. Ding et al. (2021) also reported no severe infections among 25 JDM patients receiving JAK inhibitors [11,22,27].

However, some studies have reported potential safety concerns. Le Voyer et al. (2022) described cases of herpes zoster and skin abscesses in a cohort of 10 JDM patients treated with JAK inhibitors [28]. Huang et al. (2023) identified leukopenia and cough in a subset of patients, while 39.6% were able to discontinue glucocorticoids [21]. Kostik et al. (2022) documented a case of lymphadenitis associated with tofacitinib use [29]. Quintana-Ortega et al. (2022) described a fatal outcome in an 11-year-old patient with anti-MDA5-positive juvenile dermatomyositis and rapidly progressive interstitial lung disease treated with intensive combination immunosuppression including tofacitinib, underscoring the fulminant nature and high mortality risk of this condition [30]. Despite these findings, most adverse effects were manageable, and the overall benefit–risk profile of JAK inhibitors in JDM remains favorable, particularly in refractory disease cases (Table 1).

Importantly, uncertainties regarding long-term safety persist, especially in the context of childhood growth and immune system maturation. The JAK–STAT pathway plays a role not only in immune regulation but also in cell proliferation, differentiation, and metabolic processes [31]. Prolonged inhibition during childhood therefore raises theoretical concerns related to oncological risk, endocrine development, growth patterns, and the long-term shaping of adaptive immunity [32]. To date, no increase in malignancy or severe developmental disturbances has been reported [33]; however, available studies are limited by relatively short follow-up periods.

In this case, based on World Health Organization growth standards, the patient’s weight, height, and body mass index (BMI) percentiles were around the 50th percentile at treatment initiation and remained stable over a two-year follow-up, with no clinical evidence of growth impairment. Nonetheless, this favorable individual observation does not obviate the need for long-term prospective follow-up studies and registry-based surveillance to adequately define long-term outcomes and the true safety profile of JAK inhibitors in pediatric patients.

In juvenile dermatomyositis, JAK inhibitors represent one option within advanced immunomodulatory therapies. Oral administration and avoidance of prolonged immune cell depletion may be practical advantages in younger patients. Treatment response may vary by disease phenotype, and cost considerations should be addressed on a country-specific basis. Given limited long-term safety data in children, treatment decisions should be individualized.

Management of JDM is guided by the 2017 consensus-based recommendations developed within the European SHARE initiative, which outline a standardized, stepwise approach to corticosteroid and immunosuppressive therapy, complemented by physiotherapy and close monitoring of disease activity [34]. Within this framework, emerging biomarkers—such as interferon-pathway signatures—offer an opportunity to refine treatment decisions and support the rationale for targeted therapies like JAK inhibition.

## 4. Conclusions

JAK inhibitors hold promise as targeted agents for anti-MDA5 antibody-positive juvenile dermatomyositis, with potential roles in both induction and maintenance of remission. Their ability to achieve rapid disease control supports their integration into evolving treatment algorithms; however, rigorous clinical evaluation is still needed to confirm efficacy and safety and to define evidence-based management protocols.

**Table 1:** Summary of reports on JAK inhibitor use in Juvenile Dermatomyositis (JDM).

Study	Author(s)	Year	Patient Characteristics	Treatment	Efficacy	Safety
[11]	Aeschlimann et al.	2018	13-year-old female, refractory JDM, anti-NXP2+	Ruxolitinib	Sustained remission, improved muscle strength, reduced inflammation	No major adverse events
[27]	Papadopoulou et al.	2019	11-year-old, refractory JDM	Baricitinib	Strength, skin disease improved; steroid tapering	No adverse events, relapse after stopping
[35]	Sabbagh et al.	2019	Anti-MDA5+ JDM, refractory	Tofacitinib	Muscle, skin, lung function improved	No adverse events
[36]	Melki et al.	2020	JIIM patients with/without anti-MDA5, 4 cases	Various therapies, 4 patients Ruxolitinib	Required for remission in severe skin cases	Not specified
[37]	Sozeri & Demir	2020	2 pediatric patients (JDM, refractory calcinosis)	Tofacitinib	Complete resolution of calcinosis in one patient, moderate improvement in the other	Not mentioned
[23]	Chan Ng et al.	2021	Pediatric JDM, rapidly progressive ILD	Tofacitinib	Remission achieved, ILD biomarkers improved	No adverse events
[22]	Ding et al.	2021	25 JDM patients (mean age 7.2 ± 4.0)	Tofacitinib (*n* = 7), Ruxolitinib (*n* = 18)	Rash resolved (96%), muscle strength improved (40%)	No major adverse events
[20]	Heinen et al.	2021	14-year-old male, NXP2+ JDM	Ruxolitinib	Improved strength, reduced inflammation	No major adverse events
[38]	Kim et al.	2021	4 refractory JDM cases (ages 5.8–20.7)	Baricitinib	Strength, corticosteroid tapering improved	Not specified
[39]	Quintana-Ortega et al.	2022	11-year-old, anti-MDA5+ JDM, ILD	Tofacitinib	No response	Fatal SARS-CoV-2 complication
[25]	Agud-Dios et al.	2022	5-year-old male, JDM, calcinosis, contractures	Baricitinib	Improved muscle strength, calcinosis, mobility	No major adverse events
[29]	Kostik et al.	2022	2 JDM patients (6-month follow-up)	Tofacitinib	One complete, one partial response	Lymphadenitis
[28]	Le Voyer et al.	2022	10 JDM cases (9 refractory, 1 new-onset)	Ruxolitinib (*n* = 7), Baricitinib (*n* = 3)	5 achieved inactive disease, steroids reduced	Herpes zoster, skin abscesses
[40]	Stewart et al.	2022	1 pediatric patient (JDM, MAS as initial manifestation)	IVIG, steroids, mycophenolate, anakinra, tofacitinib	Resolution of MAS, improvement in multi-organ involvement	Not mentioned
[13]	Strauss et al.	2023	4-year-old patient with MDA5 antibody	Ruxolitinib	Fast and sustained remission	No major adverse events
[21]	Huang et al.	2023	9 (previously unreported) JDM patients	Ruxolitinib (*n* = 6), Baricitinib (*n* = 3)	Rash, muscle strength, and lab markers improved; 39.6% stopped steroids	Leukopenia, cough
[24]	Kaplan et al.	2023	Anti-MDA5+ JDM, ILD, cardiac involvement	Tofacitinib	Disease control with steroid tapering	Not specified
[26]	Mastrolia et al.	2023	Refractory JDM, calcinosis	Baricitinib	Disease and calcinosis improved	Not specified
[41]	Wang et al.	2023	20 children with refractory/severe JDM	Baricitinib (*n* = 20) + steroids + immunosuppressants	95% improvement in skin rash, better muscle strength, reduced disease activity, 49% steroid reduction at 24 weeks	No serious side effects reported
[42]	Xue Y	2023	9 anti-MDA5-positive children with JDM and ILD	Tofacitinib	55.5% showed ILD improvement; 44.5% had worsened ILD; high IgG and T-cell levels correlated with poor response	Not mentioned
[43]	Zhang J	2023	A total of eighty-eight patients with JDM	Tofacitinib	Skin and muscle symptoms improved markedly. Nearly half achieved complete response, six remained on tofacitinib monotherapy. Lung disease improved in 60%, and calcinosis in 75% (complete resolution in 25%).	Only one patient had herpes zoster infection 9 months after initiation. After drug withdrawal, herpes recovered and tofacitinib was given again.
[44]	Yu et al.	2023	3 children with refractory JDM	Tofacitinib	Improved muscle strength, skin lesions, quality of life, steroid tapering	No severe adverse events
[45]	Xiangyuan C. et al.	2024	12-year-old girl with JDM who developed multiple GI perforations	Tofacitinib	Leading to gradual improvement in muscle strength and reduction in inflammation	No severe adverse events
[46]	Kinkor M et al.	2024	14-month-old female with anti-MDA5	Tofacitinib	Remission of skin and muscle disease	Not mentioned
[47]	Huang B et al.	2024	11-year-old girl with juvenile dermatomyositis (JDM), anti-MDA5 antibodies and multiple skin ulcers	Tofacitinib	At the 2-month follow-up visit, early healing of the ulcers Within 8 months, the skin ulcers healed, and muscle enzyme markers and ESR returned to normal.	Not mentioned
[48]	Bader-Meunier B et al.	2025	Thirty-nine patients with JDM	Various therapies	A significant decrease in the median Type 1 IFN score and serum IFN-α from the diagnosis of JDM to the 6-month follow-up	JAKi-related adverse events consisted of infections in nine patients (including five herpes zoster infections) and weight gain in three patients.
[49]	Arguelles Balas D et al.	2025	9-year-old Spanish boy	Tofacitinib	Remission with tofacitinib monotherapy following the failure of previous therapies	No AEs related to tofacitinib have been observed.

## Data Availability

No new datasets were generated or analyzed during the current study. Data sharing is not applicable to this article.
